# Unravelling the Identity, Metabolic Potential and Global Biogeography of the Atmospheric Methane‐Oxidizing Upland Soil Cluster α

**DOI:** 10.1111/1462-2920.14036

**Published:** 2018-01-18

**Authors:** Jennifer Pratscher, John Vollmers, Sandra Wiegand, Marc G. Dumont, Anne‐Kristin Kaster

**Affiliations:** ^1^ The Lyell Centre Heriot‐Watt University Edinburgh UK; ^2^ Institute for Biological Interfaces (IBG5), Karlsruhe Institute of Technology Karlsruhe Germany; ^3^ Department of Microbiology Institute for Water and Wetland Research, Faculty of Science, Radboud University Nijmegen The Netherlands; ^4^ Biological Sciences University of Southampton Southampton UK

## Abstract

Understanding of global methane sources and sinks is a prerequisite for the design of strategies to counteract global warming. Microbial methane oxidation in soils represents the largest biological sink for atmospheric methane. However, still very little is known about the identity, metabolic properties and distribution of the microbial group proposed to be responsible for most of this uptake, the uncultivated upland soil cluster α (USCα). Here, we reconstructed a draft genome of USCα from a combination of targeted cell sorting and metagenomes from forest soil, providing the first insights into its metabolic potential and environmental adaptation strategies. The 16S rRNA gene sequence recovered was distinctive and suggests this crucial group as a new genus within the *Beijerinckiaceae*, close to *Methylocapsa*. Application of a fluorescently labelled suicide substrate for the particulate methane monooxygenase enzyme (pMMO) coupled to 16S rRNA fluorescence *in situ* hybridisation (FISH) allowed for the first time a direct link of the high‐affinity activity of methane oxidation to USCα cells *in situ*. Analysis of the global biogeography of this group further revealed its presence in previously unrecognized habitats, such as subterranean and volcanic biofilm environments, indicating a potential role of these environments in the biological sink for atmospheric methane.

## Introduction

Methane (CH_4_) is an important greenhouse gas with a current atmospheric concentration of ∼1.84 p.p.m.v. and a global warming potential (GWP_100_) 34 times greater than CO_2_ (Ciais, 2013). Most of the atmospheric CH_4_ results from production by methanogenic archaea, the final step in anaerobic degradation of organic matter (Conrad, 2009). However, only a fraction of the CH_4_ produced is emitted to the atmosphere, the remainder is utilized by methane‐oxidizing bacteria, so‐called methanotrophs. Aerobic methane‐oxidizing bacteria generally belong to the γ‐ (type I methanotrophs), α‐ (type II methanotrophs) proteobacteria and Verrucomicrobia. The key step in aerobic methane oxidation, the initial oxidation of CH_4_ to methanol, is catalysed by the methane monooxygenase which occurs as a particulate, membrane bound form (pMMO), and as a soluble, cytosolic form (sMMO) (Hanson and Hanson, 1996). These two enzymes are distinct and are the result of two evolutionary independent processes. The *pmoA* gene, which encodes the β‐subunit of pMMO, has been successfully used as a biomarker to investigate methanotrophic communities in various environments (e.g., McDonald *et al*., 2008; Knief, 2015; Ghashghavi *et al*., 2017).

Microbial methane oxidation in soils represents the largest biological sink for atmospheric methane, with an uptake of approximately 30 Tg CH_4_ y^−1^ (6% of the global sink) (Ciais, 2013). Forest systems in particular possess the greatest atmospheric methane consumption capability of any ecosystem (Aronson *et al*., 2013). The activity of atmospheric methane uptake in these soils has shown to be sensitive to land use change and very slow to recover after disturbances of the environment (like deforestation and agricultural use) (Priemé and Christensen, 1997; Levine *et al*., 2011). Early studies in the 1990s reported Michaelis‐Menten kinetics of atmospheric CH_4_ oxidation in forest soil samples, thus establishing the activity of high affinity methanotrophs (Bender and Conrad, 1992; Bender and Conrad, 1993; Bender and Conrad, 1994). Almost all extant methanotrophs cannot grow on the low CH_4_ concentrations in the atmosphere. Several *Methylocystis* and *Methylosinus* species contain a second monooxygenase that catalyses oxidation of CH_4_ at atmospheric levels (Dunfield *et al*., 2002; Baani and Liesack, 2008; Kravchenko *et al*., 2010) but they also cannot sustain activity and growth at those low concentrations of methane (Baani and Liesack, 2008; Dunfield *et al*., 2010; Belova *et al*., 2011).

Unique clades of *pmoA* sequences in soils exhibiting atmospheric methane uptake were first reported by Holmes and colleagues (1999). A cluster they originally termed RA14 later became known as upland soil cluster α (USCα) (Knief and Dunfield 2005). Efforts to cultivate USCα, or to identify their 16S rRNA sequence, have so far been unsuccessful. Still very little is known about the identity, metabolic potential and distribution of USCα, which are proposed to be responsible for most of this atmospheric, high‐affinity CH_4_ uptake. Except for a 42 kb fosmid clone harbouring the key genes for methane oxidation (Ricke *et al*., 2005), there was so far no genomic data available for USCα: this environmentally crucial group has evaded phylogenetic identification and any cultivation or enrichment attempts since the discovery of its activity over 25 years ago (Bender and Conrad, 1992).

The fact that ecologically important microorganisms like USCα are not yet isolated imposes a further challenge on the science of soil microbial ecology. However, the development of advanced culture‐independent cell sorting and metagenomic techniques enables the further study of microorganisms in the environment. An opportunity to identify and characterize specific microbial groups from environmental samples is provided by single cell genomics and the generation of so‐called ‘mini‐metagenomes’, which couples the sorting of cells of interest from environmental samples with whole genome sequencing (Nurk *et al*., 2013; Kaster *et al*., 2014; Rinke *et al*. 2014). The information from genome analysis can then be applied to predict pathways and metabolism and design targeted isolation approaches. Biochemical markers, such as genes encoding key metabolic enzymes and ribosomal RNA (rRNA) can further be analysed with respect to phylogeny and function.

In this study, metagenomic sequencing of a forest soil with active methane uptake was used to identify the specific 16S rRNA gene sequence of USCα. Identification of the 16S rRNA gene enabled several downstream experiments that were hitherto not possible. The simultaneous fluorescent labelling of the active pMMO at low concentrations using a suicide inhibitor and the USCα specific 16S rRNA by DOPE‐FISH (Double Labelling of Oligonucleotide Probes for Fluorescence *In Situ* Hybridization) in the same cells *in situ* provided the first clear link between this cluster and the proposed uptake activity. The labelling approach was further used for targeted cell sorting and the generation of USCα‐enriched ‘mini‐metagenomes’, consisting of 10–500 cells, and was combined with four environmental metagenomes that resulted in a near complete draft genome of USCα. Analyses of the draft genome provided the first insight into the genetic and metabolic potential of USCα and its environmental adaptation strategies. The results define the USCα as a new genus within the *Beijerinckiaceae*. Using the identified USCα phylogeny, we further analysed the environmental distribution of this important cluster, compiling data from 16S rRNA gene surveys and metagenomes worldwide.

## Results and discussion

### 16S rRNA gene identity of atmospheric methane oxidizing bacteria of the upland soil cluster α (USCα) from forest soil

**Figure 1:**
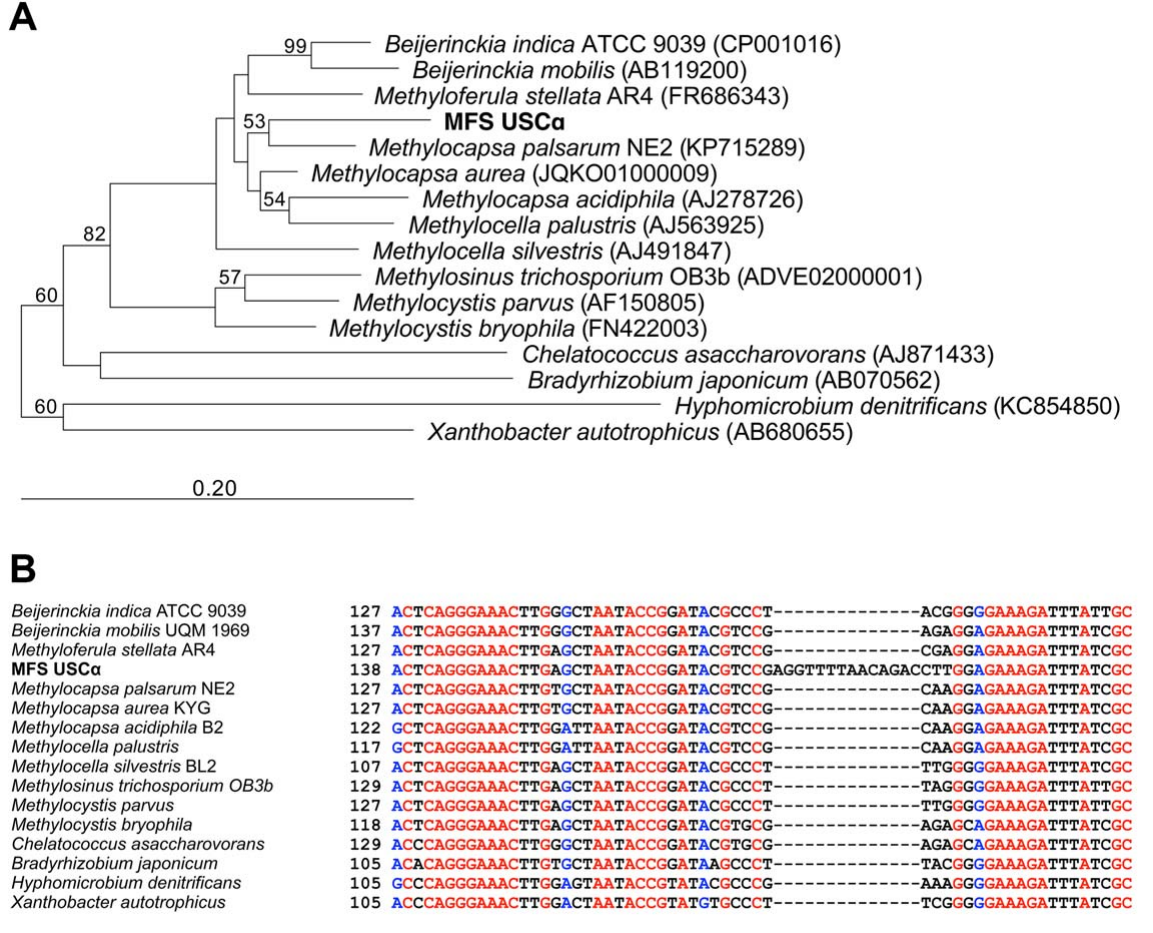
Phylogenetic analysis of the 16S rRNA gene sequence of upland soil cluster α from Marburg forest soil. **A.** Neighbor‐joining tree with Jukes‐Cantor correction of distances and 2000 bootstraps. The full 16S rRNA gene sequence from USCα (MFS USCα) soil is depicted in bold. Scale bar indicates 20% sequence divergence. **B.** Alignment of USCα 16S rRNA gene sequence with closest relatives to show sequence divergence.

Samples of an acidic (pH ∼4) forest soil from Marburg, Germany, displaying a high‐affinity CH_4_ oxidation potential (∼41 pmol g.d.w. soil^−1^ h^−1^) at atmospheric methane concentration (∼2 p.p.m.v.), were used for an initial metagenomic binning approach. USCα have shown to be the predominant methanotroph in this soil and make up ∼95% of the methanotrophic bacterial community (Pratscher *et al*., 2011). The initial metagenomic binning approach was carried out with two metagenomes from two different sampling time points (October 2013 and October 2014) based on tetranucleotide frequency (TNF) and abundance. This recovered a preliminary genome bin for USCα (completeness 57%, contamination 1.1%), with a dedicated partial 16S rRNA gene sequence. Based on this partial sequence, a specific PCR forward primer for the USCα 16S rRNA gene was designed (termed ‘MF‐1F’). This primer was paired with a universal reverse bacterial 16S rRNA primer to amplify the full 16S rRNA gene sequence of USCα, and Sanger‐sequencing of this product resulted in the reconstruction of the full‐length 16S rRNA gene sequence for phylogenetic analyses. Consistent with previous *pmoA* phylogenetic analyses (Knief *et al*., 2003; Kolb *et al*., 2005; Ricke *et al*., 2005), the USCα sequence clustered most closely to *Methylocapsa* (Fig. 1A). However, the 16S rRNA gene sequence only showed an identity of 96% to the next closest cultivated relatives, *Methylocapsa palsarum* NE2 and *Methylocapsa aurea* KYG, but is 98–99% identical to hundreds of sequences currently in databases from environmental/uncultivated organisms (see analysis of biogeography below). This phylogenetic difference was further emphasized by a 14‐bp long insert in the USCα 16S rRNA gene sequence, which is not present in any of the other cultured methanotrophs and its closest cultivated relatives (Fig. 1B).

The identification of the USCα 16S rRNA gene sequence also enabled us to reassess the relative abundance of USCα in the forest soil based on the relative abundance of its 16S rRNA gene. DNA extracts from the forest soil from October 2014 and November 2016 were used as template in 16S rRNA gene amplicon sequencing. The sequencing data not only showed that USCα indeed exhibit a relative abundance of around ∼1% of the total prokaryotic community in the forest soil but also show slight variations during different years (0.4% in October 2014 to 1.3% in November 2016). These abundances were also reflected in the respective final assembled metagenomes, showing an USCα abundance of 0.24–0.6% in October 2013 and October 2014, but an increased abundance of ∼1.7% in November 2016.

### High‐affinity methane oxidation activity linked to USCα cells *in situ*

Based on the USCα specific 16S rRNA gene sequence we designed a specific probe (termed ‘MF’) for 16S rRNA DOPE‐FISH (Double Labelling of Oligonucleotide Probes for Fluorescence *In Situ* Hybridization) (Stoecker *et al*., 2010). It has recently been reported that in some paddy soil environments, conventional methanotrophs can temporarily exhibit atmospheric methane oxidation after induction at a very high concentration of methane (Cai *et al*., 2016). This is not the case for upland forest soils, which typically do not encounter significant spikes in methane concentration from the environment resulting from endogenous methanogenesis, strongly indicating obligate high‐affinity methane oxidation in these soils. To confirm that the high‐affinity activity is indeed related to USCα, we further visualized their pMMO enzyme *in situ*, using the fluorescently labelled acetylene analogue FTCP (fluorescein thiocarbamoyl‐propargylamine) (McTavish *et al*., 1993). Acetylene acts as a suicide substrate for the methane monooxygenase enzymes (sMMO and pMMO) (Prior and Dalton, 1985) and has been shown to eliminate atmospheric methane uptake activity in forest soils (Bender and Conrad, 1992). This approach has previously been used primarily for the ammonia monooxygenase (AMO) of nitrifying bacteria, an enzyme that can also be inhibited with acetylene (McTavish *et al*., 1993; van Kessel *et al*., 2015). To evaluate this approach for methanotrophic bacteria, active cells of *Methylosinus trichosporium* OB3b (a methanotroph that possesses pMMO) and a *Methylovorus* strain (a methylotroph that does not contain pMMO or sMMO) were successfully used as positive and negative controls respectively (Supporting Information Fig. S1).

**Figure 2:**
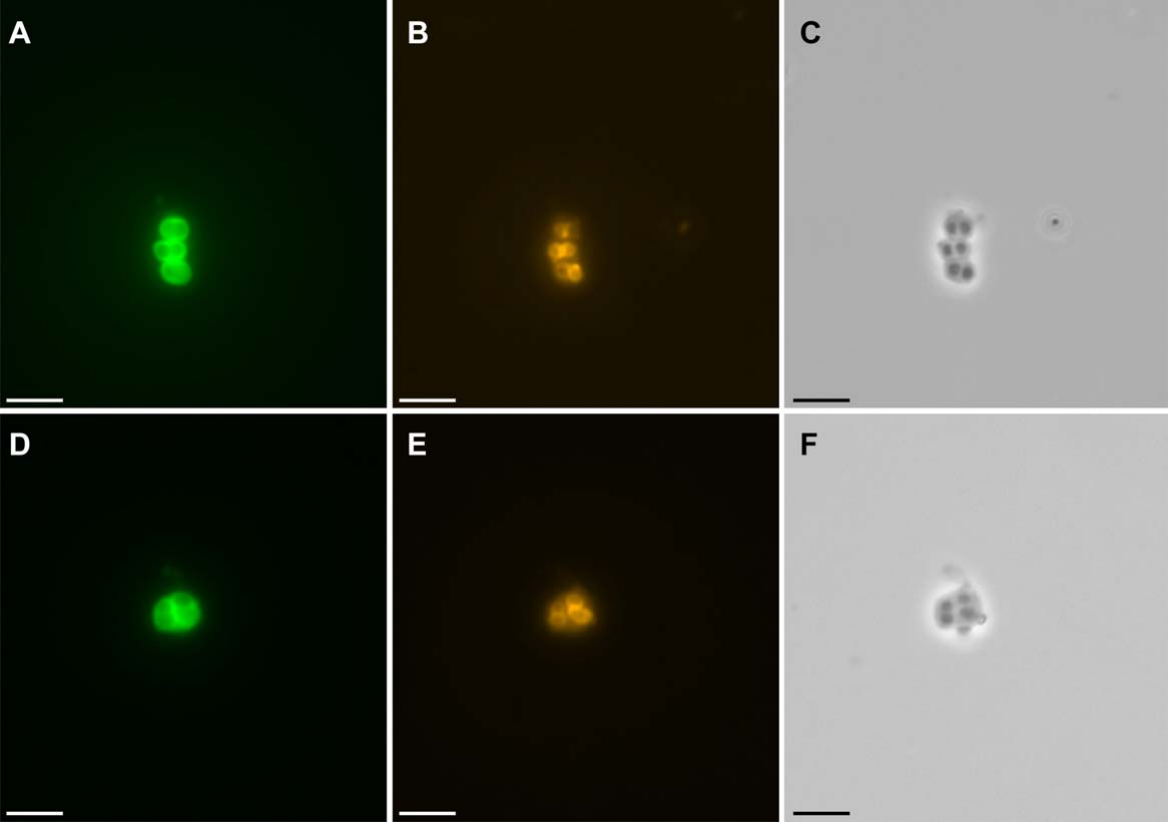
Detection of active pMMO and specific 16S rRNA in USCα cells from forest soil taking up atmospheric methane. **A** and **D**, pMMO labelling by FTCP (green), **B** and **E**, USCα specific 16S rRNA DOPE‐FISH (orange), **C** and **F**, respective phase contrast. Scale bars represent 5 μm.

We combined both 16S rRNA DOPE‐FISH and FTCP labelling to simultaneously detect and visualize USCα cells and the activity of their high‐affinity pMMO in cells extracted from the active forest soil under atmospheric and low methane concentrations (2 p.p.m.v. and 20 p.p.m.v. respectively). Cells were successfully extracted and separated from the soil using a Histodenz density gradient centrifugation method as applied for a previous *pmoA* mRNA CARD‐FISH study targeting USCα in this soil (Pratscher *et al*., 2011). Fixation‐free FISH was performed to allow for subsequent cell sorting and genome amplification (Yilmaz *et al*., 2010), and without addition of formamide to the hybridisation buffer, as use of formamide has been shown to decrease FISH signals in aerobic methanotrophs (Dedysh *et al*., 2001). Simultaneous and strong labelling of the USCα pMMO and 16S rRNA provided for the first time a direct link of the high‐affinity activity to USCα cells *in situ* (Fig. 2).

### Draft genome of USCα from forest soil recovered by a combination of targeted cell enrichments and metagenomic data

Except for a 42 kb fosmid clone from the forest soil used in our study, harbouring the *pmoCAB* genes (Ricke *et al*., 2005), there is no genomic data currently available for the USCα. From previous studies using quantitative PCR for the *pmoA* gene, USCα is estimated to have a relative abundance of ∼1% in these highly diverse soils (Knief *et al*., 2003), and we were able to confirm this estimation in this study using 16S rRNA gene amplicon sequencing as described above. To overcome this low abundance, we combined targeted fluorescence *in situ* hybridization–fluorescence activated cell sorting (FISH‐FACS) for USCα‐enriched metagenomes with direct metagenomic sequencing from forest soil. The double labelling approach of active pMMO and specific 16S rRNA in USCα cells extracted from the forest soil was used to enable FISH‐FACS (Yilmaz *et al*., 2010). Labelled cells were sorted by fluorescent activated cell sorting (FACS) in 384 well plates, each well containing a defined number of cells. After the separation, the cells were lysed to release their DNA. Multiple displacement amplification (MDA) was used to amplify the DNA to a sufficient amount for downstream applications. PCR was used to screen for *pmoA* and 16S rRNA genes after the amplification. PCR and Sanger Sequencing resulted in four wells containing ‘mini‐metagenomes’ of 10–500 cells enriched in USCα cells. In addition, we deeply sequenced another 2 metagenomes from the forest soil for a total of 4 metagenomes from different years (2013, 2014, 2016) and with different sequencing strategies (see Experimental Procedures). The sequencing approach resulted in a total data volume of ∼70 Gb.

Combining all of this data from the (mini‐)metagenomes for a targeted binning strategy led to the recovery of a nearly complete genome for USCα (Table 1). Assembled contigs were pre‐partitioned based on relative abundance and then binned using a combination of compositional as well as differential coverage approaches. Subsequent bin refinement was based on taxonomic classification as well as an additional differential coverage approach (see Supporting Information and Supporting Information Table 1).

**Table 1:** Summary statistics of the USCα genomic bin.

Bin size (Mb)	3.71
Number of contigs	239
N50 value	35776
Completeness (%)	86
Contamination (%)	0.81
GC content (%)	59.8
Number of predicted CDS	3889
Percentage of ‘hypothetical proteins’	45%
Median coverage in MFS1	2.6×
Median coverage in MFS2	2.9×
Median coverage in MFS3_1	22×
Median coverage in MFS3_2	32×

The USCα draft genome has a size of 3.71 Mb in 239 contigs, an estimated completeness of 86% with a low estimated contamination of 0.81% and a GC content of 59.8%. These can be considered excellent binning results because genomes of *Beijerinckiaceae* have been shown to contain genomic islands of varying codon usage/tetranucleotide frequency patterns, thought to originate from lateral gene transfer events (Tamas *et al*., 2014), which pose a challenge for binning. The GC content and genome size of the USCα draft genome are in line with most methanotrophic *Beijerinckiaceae* (Tamas *et al*., 2014; Miroshnikov *et al*., 2017).

**Figure 3:**
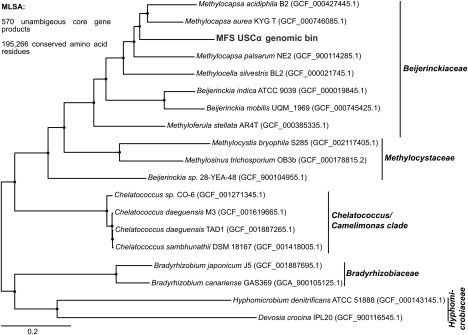
Phylogenetic tree showing the placement of the USCα genomic bin within the *Beijerinckiaceae*. Approximate maximum likelihood tree generated using FastTree (Price *et al*., 2010) based on multilocus sequence analyses (MLSA) of conserved 195 266 amino acid positions from 570 concatenated unambiguous core gene products. For each reference genome, the respective NCBI assembly accession number is given in parentheses. The USCα genomic bin is marked in red, and the respective bin designation is given in parentheses. Shimodaira‐Hasegawa support values were >99 at all nodes.

A 16S rRNA gene sequence matching the identified 16S rRNA gene sequence from the preliminary metagenomic assembly and subsequent PCR and Sanger sequencing (Supporting Information Fig. S2) was also reconstructed by the assemblies and was associated to the USCα genomic bin due to phylogenetic congruency, matching abundance profiles as well as co‐occurrence within USCα‐enriched mini‐metagenomes. Phylogenomic placement of this bin compared with known reference genomes based on multilocus sequence analyses (MLSA) further supported the positioning of this cluster close to *Methylocapsa* (Fig. 3). Qin *et al*. proposed a new metric for delineating existing and defining novel prokaryotic genera: the percentage of conserved proteins (POCP) (Qin *et al*., 2014). A genus is thereby defined as a group of species with POCP values above 50% between all members. Accordingly, a novel genus should display POCP values below 50% to any strain of all related existing genera. The basic applicability of this metric for *Rhizobiales* could be confirmed by analyses of respective reference genomes (Supporting Information Fig. S3). As the USCα genomic bin displayed POCP values below 44% to all other *Beijerinckiaceae* reference genomes, including the most closely related *Methylocapsa* strains, it is likely that this bin represents a novel uncultured genus. This interpretation is additionally supported by the 14‐bp USCα 16S rRNA signature gene insert, which was found to be absent in all related *Beijerinckiaceae*, including all *Methylocapsa* strains. For these reasons, we hereby propose the classification of the first high quality USCα genomic bin as *Candidatus* ‘Methyloaffinis lahnbergensis’ gen. nov, sp. nov. [N.L. n. *methyl* the methyl group; L. n. *affinis* affinity (connected with); L. adj, *lahnbergensis* from Lahnberge (near Marburg, Germany)]

### Metabolic reconstruction of the USCα draft genome

**Figure 4:**
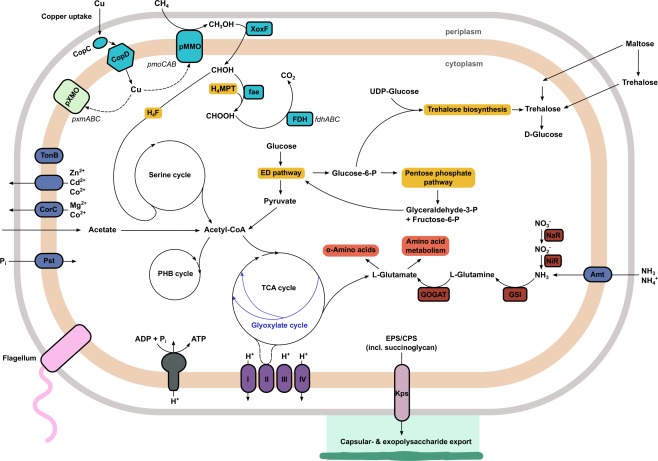
Reconstruction of methanotrophic and central metabolism pathways in USCα. The methanotrophy‐related pathways are highlighted in teal. Abbreviations: CoA, coenzyme A; ED pathway, Entner‐Doudoroff Pathway; EPS/CPS, exopolysaccharides/capsular polysaccharides; fae, formaldehyde activating enzyme; FDH, formate dehydrogenase; GOGAT, glutamate synthase; GSI, glutamine synthetase; H_4_F, tetrahydrofolate; H_4_MPT, tetrahydromethanopterin; MDH, methanol dehydrogenase; NaR, nitrate reductase; NiR, Nitrite reductase; PHB, polyhydroxybutyrate; P_i_, inorganic phosphate; pMMO, particulate methane monooxygenase; TCA cycle, tricarboxylic acid cycle.

Metabolic reconstruction of the USCα draft genome confirmed the expected methanotrophic lifestyle (Fig. 4). USCα exhibited only one copy of the pMMO (*pmoCAB*) and no sMMO, thus providing further evidence that this pMMO enzyme is responsible for the high‐affinity methane oxidation activity. Genes encoding CopCD proteins were identified, which have an important role in copper homoeostasis in some bacteria (Lawton *et al*., 2016). Copper is essential for pMMO activity, but it should be noted that copper homoeostasis appears to be controlled by multiple systems in *Methylosinus trichosporium* OB3b as CopCD was found not to be essential for copper uptake (Gu *et al*., 2017). Although we cannot rule out the presence of uncharacterized copper transport proteins in USCα, no genes related to the specific methanobactin (mb) copper‐binding proteins (Semrau *et al*., 2010; DiSpirito *et al*., 2016) or the recently discovered copper storage proteins (Csp) could be identified (Vita *et al*., 2015). The USCα genome further contained several genes encoding postulated copper metallochaperones. The draft genome also contained the genes for a pXMO (encoded by *pxmABC*), a pMMO/AMO‐related particulate Cu‐monooxygenase of unknown function, speculated to enable the utilization of other substrates or detoxification reactions (Tavormina *et al*., 2011; Knief, 2015). This gene showed a nucleotide identity of only 76% to the closest related *pxmA* sequence from *Methylocystis bryophila* strain S285. The second step in the methane oxidation pathway, the oxidation of methanol, was identified by the presence of genes encoding a PQQ‐dependent XoxF‐type methanol dehydrogenase (Keltjens *et al*., 2014). No MxaF‐type methanol dehydrogenase was found. The XoxF enzymes require lanthanides for activity and expression (Chistoserdova 2016; Krause *et al*., 2017) and with XoxF as the only methanol dehydrogenase in the genome draft, this finding suggests the dependence of USCα on lanthanides for growth and activity.

Instead of a formaldehyde dehydrogenase, the USCα genome possessed a gene encoding formaldehyde activating enzyme (fae), which is a common pathway in methylotrophs (Vorholt *et al*., 2000). The potential for formate oxidation was presented by formate dehydrogenase genes. The USCα draft genome encoded all necessary pathways for a microorganism with a C_1_ metabolism, including C_1_ unit incorporation in the serine cycle via tetrahydrofolate (H_4_F), and C_1_ transfer via tetrahydromethanopterin (H_4_MPT).

In addition, it contained pathways for the utilization of other carbon sources such as acetate (via the glyoxylate cycle). This supports the findings of our previous research, using a ^13^C‐labelled acetate stable isotope probing (SIP) approach, that USCα are facultative, rather than obligate methanotrophs (Pratscher *et al*., 2011). The gene encoding isocitrate lyase for the glyoxylate cycle was not recovered, but because no ethylmalonyl‐CoA pathway could be proposed from the genome as an alternative, the missing isocitrate lyase gene can be attributed to the genome draft not being 100% complete rather than a non‐functional glyoxylate cycle. Closely related methanotrophs, such as *Methylocapsa acidiphila* and *Methylocapsa palsarum* also do not possess an ethylmalonyl‐CoA pathway but a glyoxylate cycle (Dedysh *et al*., 2002; Miroshnikov *et al*., 2017). The USCα draft genome contained the complete set of genes for the tricarboxylic acid cycle and also exhibited a full Entner‐Doudoroff pathway (Kalyuzhnaya *et al*., 2015). It did not seem to possess the full set of genes for the Calvin‐Benson‐Bassham cycle, in contrast to other *Beijerinckiaceae* methanotrophs (Miroshnikov *et al*., 2017). Genes involved in N_2_ fixation could not be identified, unlike *Methylocapsa acidiphila* and *Methylocapsa palsarum*. Complete pathways were recovered for polyhydroxybutyrate (PHB) and polyphosphate metabolism. USCα were found to have a relative abundance of ∼1% in soil, but we assume that they are oligotrophic and reach this abundance slowly. This is consistent with their long recovery times in soil following disturbance (e.g., Levine *et al*., 2011). An oligotrophic lifestyle is exemplified by their apparent affinity for atmospheric methane. Also, we know that they can use acetate, which is typically low in forest soils (Fox and Comerford, 1990). Furthermore, we do not detect genes for nitrogen fixation, suggesting they are adapted to scavenging soil nitrogen, which is typically scarce.

We were further able to identify genes involved in the production of exopolysaccharides (EPS), including succinoglycan, an acidic EPS contributing in the formation of moisture‐retaining biofilms (Schmid *et al*., 2015). Genes identified for this process were, for example, exopolysaccharide production regulator ExoR and production protein ExoZ. *Methylocapsa acidiphila* is known to form exopolysaccharides when grown under acidic conditions on nitrogen‐free media (Dedysh *et al*., 2002). In addition to this, we also found genes involved in the biosynthesis of trehalose, including trehalose synthase, trehalose‐6‐phosphate phosphatase, alpha,alpha‐trehalose‐phosphate synthase and glucoamylase. Trehalose is a non‐reducing disaccharide that acts as a storage carbohydrate and most effectively protects membranes against dehydration (Reina‐Bueno *et al*., 2012). Soil hydrophobicity is a known occurrence in forest soils (Buczko *et al*., 2006), so these desiccation protection mechanisms provide valuable insights into the growth behaviour and environmental adaptation strategies of this organism. Soil with a low water content presents a trade‐off situation for methanotrophic bacteria, as it enables faster and better diffusion of methane and oxygen through the soil, increasing atmospheric methane uptake capacity, but on the other hand this imposes enhanced osmotic stress on the microbial cells, potentially leading to slow growth or the formation of resting cells (Whittenbury *et al*., 1970; Eller and Frenzel, 2001). Atmospheric CH_4_ oxidation activity in forest soils is fragile and heavily impacted by land use and changing soil conditions. For example, with soils in Denmark and Scotland, over 100 years were necessary to recover precultivation rates of atmospheric CH_4_ uptake after land‐use change from agriculture to woodland (Priemé and Christensen, 1997). Forest clear‐felling and deforestation have also been reported to turn soils from being a sink for CH_4_ into a net source (Keller *et al*., 1990; Keller and Reiners, 1994; Zerva and Mencuccini, 2005). If USCα require growth in biofilms, this might provide a potential explanation for their slow growth and decreasing presence in case of soil disturbance. The information recovered from the draft genomes also presents the opportunity to be used for a more targeted isolation approach for USCα. Assays for isolation of biofilm‐forming bacteria have been reported (Dedysh, 2011). The application of these assays (under slightly acidic conditions) could be combined with the use of additional carbon sources for heterotrophic growth (such as acetate) and the supplementation of various copper and lanthanide concentrations, required for the pMMO and XoxF respectively.

### First environmental survey of USCα methanotrophs based on 16S rRNA shows some unexpected occurrences

**Figure 5:**
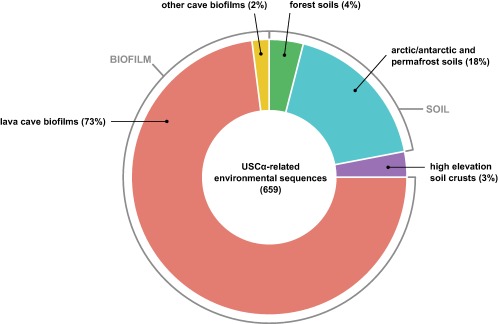
Environmental database survey of USCα on 16S rRNA gene level. Distribution of hits by source habitat of environmental 16S rRNA gene sequences ≥ 98% identical to USCα (obtained by BLAST). Number in brackets indicates total number of sequence hits. A generated RAxML tree clustered the environmental sequences with the full USCα 16S rRNA sequence with 100% bootstrap support.

Building on the discovery of the USCα 16S rRNA gene sequence, we were able to analyse for the first time their global biogeography by compiling public data from 16S rRNA gene surveys and metagenomes worldwide. So far, the *pmoA* sequence of USCα was the only available identifier, meaning that USCα could never be analysed and recognized in 16S rRNA gene based analyses. Here, we used the USCα specific 16S rRNA gene sequence as a query against the NCBI nr database. This survey returned around 660 sequences from a wide variety of environments with 99–98% identity to the USCα sequence from the Marburg forest soil (Fig. 5). Hits from this search confirmed their presence and associated activity in forest soils (such as mixed, trembling aspen, bamboo, pine and montane forests) and Antarctic and permafrost soils (Martineau *et al*., 2014). USCα 16S rRNA gene sequences were further identified in datasets from more ‘unusual’ environments, such as alpine grasslands and glacier forefields. Surprisingly, in addition to already known habitats for USCα, we also found the presence of this cluster in a variety of subterranean and cave biofilm environments, including subterranean granite, marble caves and lava tubes. USCα specific 16S rRNA gene sequences not only detected in cave biofilms and microbial mats came mostly from environments of volcanic origin (such as from Terceira and Pico, Azores, Portugal; Pico del Aguila, Mexico; Lava Beds National Monument, CA, USA; Hnausahraun lava flow, Iceland; Kipuka Konohina Cave System, Hawaii, USA) but also from limestone and marble caves (e.g., Roraima Sur Cave, Venezuela, and Oregon Caves National Monument, USA). In some of the cave population datasets (e.g., from Portugal), the USCα sequences even exhibited a considerably higher relative abundance of up to 10% of the total microbial community, indicating not only the presence but also potential key role of USCα in these environments. There have been very few indications for the presence of atmospheric methane oxidation, for example, in volcanic soil environments on Hawaii and andisols on Tenerife (King and Nanba, 2008; Maxfield *et al*., 2009), and it was so far postulated that the atmospheric methanotrophs are dependent on vegetated ecosystems with significant soil accumulation (King and Nanba, 2008) (hence the name ‘upland soil’ cluster). The unexpected presence of USCα 16S rRNA gene sequences in datasets from cave wall biofilms and subterranean ecosystems around the world (Hawaii‐USA, Azores/Portugal, Tenerife/Spain, USA, Venezuela, Mexico, China, Iceland, Korea, Slovenia, Bosnia and Herzegovina) show that this might not be accurate, further supported by the presence of biofilm‐related capacities in the USCα draft genome. Cave systems may be able to provide a favourable environment for atmospheric methane oxidizers and their potential for growth in biofilms. Cave systems can offer a constant supply of methane, a stable climate and high copper bioavailability (Northup *et al*., 2011). Our findings are further supported by a recent study that reported seasonal total methane depletion in limestone caves from Australia and the presence of methanotrophic bacteria in those caves using *pmoA* gene diversity analysis (Waring *et al*., 2017). We also found the presence of the specific 16S rRNA gene sequence related to the upland soil cluster gamma (USCγ) in a large number of the same cave datasets. USCγ was just very recently identified to belong to the *Chromatiales*, based on a draft genome (Edwards *et al*., 2017).

All of these findings strongly suggest a potential unrecognized role of these previously unnoticed environments in the biological sink for atmospheric methane and might lead to a reassessment of methane sinks around the globe. The identification of the 16S rRNA gene sequence will also lead to an easier and faster method for future screening, monitoring and quantification of USCα in the environment, in order to determine their response to climate or land use change.

## Conclusion

In this study, we applied a combination of metagenomic sequencing, targeted cell sorting and phylogenomic and biogeography analyses to unravel the identity of the atmospheric methane‐oxidizing bacteria of the USCα clade from forest soil and connect it to their proposed activity, investigate their genetic potential and re‐assess their environmental distribution. The distinctive 16S rRNA gene sequence from USCα contains a 14‐bp long insert not present in any of the cultured relatives but in a significant number of environmental sequences. Comparative analyses of the 16S rRNA gene sequences and the USCα genome bin identified this cluster as a new genus in the *Beijerinckiaceae*, close to *Methylocapsa*, for which we propose the name of *Candidatus* ‘Methyloaffinis lahnbergensis’. Simultaneous fluorescent labelling of the active pMMO and the specific 16S rRNA in USCα cells *in situ* further revealed a definite connection between their identity and proposed activity. The recovered draft genome provides important insights into the metabolic properties and adaptation of this group. No attempt to isolate a representative of the USCα clade has been successful so far, yet isolation remains an important next step in describing this environmentally crucial group. Obtaining an isolate will allow the high‐affinity methane oxidation ability to be confirmed. In addition, it will enable experiments to determine the physiological and biochemical basis for oligotrophic growth on methane as well as other key physiological characteristics of USCα, such as their sensitivity to environmental disturbance. The genome information and metabolic potential presented in this study now offers the opportunity to be exploited for more targeted isolation approaches. Re‐assessment of the biogeography of USCα significantly expanded our knowledge of their environmental distribution, giving the first indications for potential activity in unexpected environments. The results from this study can now be applied to improve future detection, monitoring and quantification of USCα in environments worldwide and to follow and predict the responses of this important methane sink to changing environmental conditions.

## Experimental procedures

### Soil sampling

Soil was sampled using 10 cm long soil cores in October 2013, October 2014 and November 2016 from a forest soil in Marburg, Germany. The field site and soil properties were described previously (Knief *et al*., 2003; Kolb *et al*., 2005; Pratscher *et al*., 2011). The soil was homogenized and stored at 4°C until further use. The rate of oxidation of ambient (∼ 2 p.p.m.v.) CH_4_ was measured by gas chromatography over a 24 h period using 10 g of soil in sealed 120 ml serum bottles.

### Nucleic acid extraction from soil and metagenome sequencing

Nucleic acids were extracted from soil using a hexadecyltrimethylammonium bromide (CTAB)‐based protocol (Griffiths *et al*., 2000) with minor modifications. Soil (0.5 g) were added to a tube of Lysing matrix E beads (MP Biomedicals UK) and mixed with, 0.5 ml of 6% CTAB extraction buffer and 0.5 ml phenol chloroform isoamyl alcohol (25:24:1) in 2.0 ml screw‐cap tubes. Cells were lysed in a FastPrep instrument (MP Biomedicals UK) for 30 s at 5.5 m s^−1^ and supernatants were extracted twice using phenol chloroform isoamyl alcohol (25:24:1) and chloroform isoamyl alcohol (24:1). Nucleic acids were precipitated with polyethylene glycol (PEG) 6000 solution (30%) and dissolved in 100 ml of nuclease free water (Ambion, Thermo Fisher Scientific).

A total of four metagenomes were generated from the DNA extractions. Those four metagenomes were labelled MFS_1 (October 2013), MFS_2 (October 2014) and MFS_3_1 and MFS_3_2 (both November 2016). MFS_1 was sequenced at Genewiz (South Plainfield, NJ, USA) using Illumina HiSeq2500 with 2 × 100 paired end cycles and resulted in 17.1 Gb of sequence data. MFS_2 was sequenced at the DSMZ (Braunschweig, Germany) using an Illumina MiSeq with 2 × 300 paired‐end cycles and resulted in ∼12 Gb sequence data. MFS_3_1 and MFS_3_2 were both sequenced using an Illumina NextSeq with 2 × 150 paired end cycles, resulting in ∼18 and ∼23 Gb of sequencing data respectively.

### PCR of USCα specific 16S rRNA gene

The USCα specific 16S rRNA gene primer was designed using the ARB software package (Ludwig, 2004) based on the partial 16S rRNA gene sequence recovered from the preliminary metagenome binning approach. The 20‐bp long primer was named MF‐1F (GAGGTTTTAACAGACCTTGG). For identification of the full‐length USCα 16S rRNA gene sequence, the 16S rRNA gene from the Marburg forest soil was amplified from the DNA extractions using the designed forward primer MF‐1F (this study) and the general bacterial reverse primer Eub1392R (Amann *et al*., 1995). The PCR temperature profile consisted of an initial denaturation and 25 cycles of denaturation, annealing and extension at 94, 55 and 72°C for 30, 45 and 60 s, respectively, followed by a final extension step at 72°C for 10 min. The purified PCR product was sequenced by Eurofins Genomics (Munich, Germany) and a phylogenetic tree was reconstructed from the sequence data using the ARB software package (Ludwig, 2004). For this, the tree was inferred by neighbor‐joining algorithm with Jukes‐Cantor correction of distances using 2000 bootstrap replicates and was verified with a tree calculated using maximum likelihood.

### 16S rRNA gene amplicon sequencing for forest soil

To generate amplicons of the 16S rRNA gene from DNA extractions from Marburg forest soil from October 2014 and November 2016, the primer set 515F/806R of the V4 variable region of the 16S rRNA gene (Caporaso *et al*., 2012) was used. After amplification by PCR, amplicon sequencing was performed on an Illumina MiSeq system (MR DNA, Shallowater, TX, USA) followed by sequence analysis and phylogenetic classification using QIIME (Caporaso *et al*., 2010).

### FTCP labelling of active USCα pMMO and DOPE‐FISH of USCα 16S rRNA

Cells from the forest soil incubated for 2–3 days with 2–20 p.p.m.v. CH_4_ were extracted using a Histodenz density centrifugation method as described previously (Pratscher *et al*., 2011). This method provides an extraction efficiency of up to 75% (Caracciolo *et al*., 2005) and has been shown to recover active atmospheric methane oxidizers from soils (Amaral *et al*., 1998; Pratscher *et al*., 2011). An additional separation step was added by sonicating the soil mixtures for 10 min in a sonicating water bath prior to centrifugation. Extracted cells were washed three times with PBS. Cells not used for direct FTCP labelling were then resuspended in 1× PBS, 10% (v/v) of a glycerol‐TE solution was added to the cells, the cell solution was incubated for 1 min and then stored at −20°C until further processing.

For FTCP labelling of active pMMO, cells extracted from the active forest soil were immediately resuspended in 50 mM NaPO_4_ buffer (pH 7.5) and incubated for 2 h at room temperature on a shaker at 150 r.p.m. with freshly prepared fluorescein thiocarbamoylpropargylamine (FTCP) (McTavish *et al*., 1993; van Kessel *et al*., 2015). After incubation, cells were harvested by centrifugation, washed twice with 1× phosphate buffer saline (PBS), resuspended in 1 ml 1× PBS/10% glycerol and stored at −20°C until further processing for DOPE‐FISH. To evaluate this approach for methanotrophic bacteria, active cells of *Methylosinus trichosporium* OB3b (a methanotroph that contains pMMO) and a *Methylovorus* strain (a methylotroph that does not contain pMMO or sMMO) were successfully used as positive and negative control respectively (Supporting Information Fig. S1).

The USCα specific 16S rRNA DOPE‐FISH probe was designed using the ARB software package (Ludwig, 2004). The probe was named ‘MF’ and labelled with a Cyanine3 fluorochrome at both the 3′ and 5′ end (Cy3‐CCAAGGTCTGTTAAAACCTC‐Cy3) (purchased from Sigma‐Aldrich, UK). To generate positive controls for 16S rRNA DOPE‐FISH, the 16S rRNA gene of USCα from the forest soil was cloned into *E. coli* Top10 competent cells using primers MF‐1F (this study) and 1392R (Amann *et al*., 1995) and expressed using vector pBAD as previously described by Pernthaler and Amann (2004). Clones were fixed in 2% (v/v) formaldehyde for 30 min at room temperature, centrifuged and washed once with 1× PBS and twice with 50% ethanol in PBS. Non‐induced clones and cells of *Methylosinus trichosporium* OB3b were successfully used as negative controls (Supporting Information Fig. S1). The hybridization and detection procedures were based on the protocol described by Yilmaz and colleagues (2010), with some minor modifications. Three hundred microliter of cell suspension extracted from the forest soil and labelled with FTCP (see above) were added to a 1.5 ml centrifuge tube and washed three times with 1× PBS. Cells were then subjected to an ethanol dehydration series (50%, 80%, 98% EtOH, 3 min incubation each). Hybridisation was performed for 3 h at 34°C using a formamide concentration of 0% in the hybridisation buffer. After the post‐hybridisation washing steps, 5–10 μl of labelled cells were applied to wells of a Teflon‐coated glass slide and mounted with the antifading agent Citifluor AF1 (Citifluor, London, UK). Hybridization preparations were visualized by fluorescence microscopy (Axiophot; Carl Zeiss Microimaging GmbH). Labelled cells were then stored in 10% glycerol at −80°C until cell sorting.

### Targeted cell sorting of double‐labelled USCα cells

Targeted prokaryotic cell enrichments were performed using fluorescence activated cell sorting (FACS), followed by chemical lysis and multiple displacement amplification (MDA) of DNA. To sort the USCα cells extracted from the active forest soil and double labelled with FTCP and 16S rRNA DOPE‐FISH (see above), the cell sample was thawed on ice, filtered via gravity flow filtration using a polycarbonate filter with 10 μm pore size (Celltrics filter, Partec, Münster, Germany) to eliminate larger particles that could block the FACS machine, and sonicated in a sonicating water bath for 3–5 s. Depending on the cell concentration/density determined by FACS, the sample was further diluted with 1× PBS. Unlabelled extracted cells from the forest soil were used as baseline control for gating of the labelled populations in the double‐labelled sample and to exclude as many auto fluorescent cells as possible. Defined numbers of cells were then sorted into a 384 well plate. Cell lysis and MDA were performed using the REPLI‐g Single Cell kit (Qiagen, Germany). In order to determine whether DNA was amplified in the reaction, SYTO® 13 was added to the reaction mixtures. After the master mix was added, the 384 well plate was thermo‐sealed with adhesive films and an ALPS™25 Manual Heat Sealer and incubated in a CFX‐384 Real‐Time System Thermal‐Cycler (Bio‐Rad Laboratories, Inc, California, USA) for 8 h. After incubation, the plates were stored at −25°C until further processing. DNA quantifications of MDA products were performed using the Qubit^®^ 3.0 Fluorometer (Thermo Fisher Scientific Inc, Massachusetts, USA) and the Qubit^®^ dsDNA HS Assay Kit (Thermo Fisher Scientific Inc, Massachusetts, USA). For quantification, the MDA products were diluted 1:20.

MDA products were screened for the USCα specific 16S rRNA and *pmoA* gene using targeted PCR with primer set MF‐1F/Eub1392R (protocol as described above) and A189f/Forest675r (Kolb *et al*., 2003) respectively. A total of four wells enriched in USCα cells were then selected for sequencing. Amplified DNA was again quantified using a Qubit 1.0 fluorometer and a dsDNA HS assay kit (Life Technologies, Darmstadt, Germany). Libraries were constructed using a NEB NextUltra DNA Library Preparation Kit (NEB), following manufacturer's instructions. Sequencing was performed on an Illumina MiSeq machine with paired end settings and 301 cycles per read.

### Bioinformatic analyses of mini‐metagenomes and full metagenomes

See Supporting Information.

### Phylogenetic analyses of the USCα biogeography

16S rRNA gene sequences with nucleotide identities ≥ 98% to the 16S rRNA gene sequence of USCα were identified in the NCBI nr database using BLAST. 16S rRNA sequence hits were imported into the SILVA RNA database. Sequence alignments where generated and manually curated, and a phylogenetic tree was reconstructed from the sequence data using the ARB software package (Ludwig, 2004). For this, the tree was inferred using RAxML with 100 replicates.

## Conflict of Interest

The authors declare no competing interests.

## Author contributions

JP and AK designed research. JP performed research. JP, JV and SW analysed data. JP wrote the manuscript with input from all co‐authors.

## Sequence data deposition

The USCα 16S rRNA gene sequence and draft genome have been deposited at GenBank/ENA/DDBJ under accession nos. MG203879 and PEFW00000000, respectively.

## Supporting information

Additional Supporting Information may be found in the online version of this article at the publisher's web‐site:

**Fig. S1.** Controls for 16S rRNA DOPE‐FISH and pMMO labelling. **a**, USCα specific 16S rRNA DOPE‐FISH (with Cy3 labelled probe MF1) with induced *E.coli* top10 clone expressing the USCα 16S rRNA gene as positive control (a), and non‐induced clone (c) and cells of *Methylosinus trichosporium* OB3b (e) as negative controls. b, d, f are respective phase contrasts. Scale bars represent 10 μm. **b,** pMMO labelling by FTCP with active cells of *Methylosinus trichosporium* OB3b as positive control (a) and *Methylovorus* cells as negative control (c). b and d are respective phase contrasts. Scale bars represent 5 μm.
**Fig. S2. a**, Partial view of 16S rRNA gene alignments showing a short insertion within the USCα 16S rRNA gene and divergence to related *Rhizobiales* sequences. The bottom histogram indicates the conservedness of each alignment position within the comparison strains. **b**, Phylogenetic tree showing the placement of the USCα associated 16S sequences within the *Beijerinckiaceae*. Neighbor‐joining tree generated using Arb (Ludwig, 2004) based on 1267 shared alignment positions. For each reference sequence, either the respective NCBI assembly accession number or the genome assembly accession number followed by the respective locus tag are given in parantheses. USCα associated sequences are marked in red. Bootstrap support values are indicated by colour at each node. *Escherichia coli* KCTC 2441 (accession: EU014689) served as distant outgroup and root (not shown).
**Fig. S3.** Pairwise pocp values. Values above the proposed genus cutoff (Qin *et al*., 2014) are indicated in red.
**Fig. S4.** Alignment of metagenomic contigs against the USCα fosmid reference CT005232 (Ricke *et al*. 2014), prior to reassembly of the USCα genomic bin. Annotated ORFs are represented as arrows, colourcoded as indicated by the legend included on the lower left. Subsequences which were aligned between contigs are shown connected by coloured blocks, which are colourcoded based on sequence identity. The USCα Fosmid reference could be almost completely reconstructed by only three contigs (mergemg123_101251, mergemg123_100323 and mergemg123_128398) showing >99.9% sequence identity over the complete respective alignment length as well as compatible coverage profiles. In addition, the fosmid reference sequence context was expanded by ∼ 38 kb by metagenome contig mergemg123_100323. A similar expansion of the 5′‐end of the fosmid reference was not possible, due to the presence of a transposase gene, The USCα signature pmoBAC genecluster was found to be encoded contig mergemg123_128398 as well as contig mid_NODE_24546, sharing 100% sequence identity over the gene cluster but differing at the extreme contig ends, thereby indicating potential strain heterologies concerning the exact genomic location of this gene cluster. However, in contrast to contig mergemg123_128398, the coverage profile of contig mid_NODE_24546 did not prove compatible with the fosmid reference and the respective genomic bin, therefore this contig was excluded from the metagenomic bin.

**Table S1.** Basic bin quality metrics and taxonomic placement of metagenomic bins. The bin quality metrics ‘Completeness’, ‘Contamination’ and ‘Strain heterogenity’ were calculated using the ‘Lineage workflow’ of CheckM (Parks *et al*. 2015) during different bin processing steps. However, read exctraction and reassembly was only performed for the USCalpha genomic bin (highlighted in yellow). A brief explanation of each metric is given in the footnotes below the table.
