# Supplementary material for: Unravelling the Identity, Metabolic Potential and Global Biogeography of the Atmospheric Methane‐Oxidizing Upland Soil Cluster α
Source: Environ Microbiol. 2018 Jan 18;20(3):1016–29. doi: 10.1111/1462-2920.14036 (PMC6849597; doi:10.1111/1462-2920.14036)
Supplement: Supplementary file 1 — Fig. S1. Controls for 16S rRNA DOPE‐FISH and pMMO labelling. a, USCα specific 16S rRNA DOPE‐FISH (with Cy3 labelled probe MF1) with induced E.coli top10 clone expressing the USCα 16S rRNA gene as positive control (a), and non‐induced clone (c) and cells of Methylosinus trichosporium OB3b (e) as negative controls. b, d, f are respective phase contrasts. Scale bars represent 10 μm. b, pMMO labelling by FTCP with active cells of Methylosinus trichosporium OB3b as positive control (a) and Methylovorus cells as negative control (c). b and d are respective phase contrasts. Scale bars represent 5 μm. Fig. S2. a, Partial view of 16S rRNA gene alignments showing a short insertion within the USCα 16S rRNA gene and divergence to related Rhizobiales sequences. The bottom histogram indicates the conservedness of each alignment position within the comparison strains. b, Phylogenetic tree showing the placement of the USCα associated 16S sequences within the Beijerinckiaceae. Neighbor‐joining tree generated using Arb (Ludwig, 2004) based on 1267 shared alignment positions. For each reference sequence, either the respective NCBI assembly accession number or the genome assembly accession number followed by the respective locus tag are given in parantheses. USCα associated sequences are marked in red. Bootstrap support values are indicated by colour at each node. Escherichia coli KCTC 2441 (accession: EU014689) served as distant outgroup and root (not shown). Fig. S3. Pairwise pocp values. Values above the proposed genus cutoff (Qin et al., 2014) are indicated in red. Fig. S4. Alignment of metagenomic contigs against the USCα fosmid reference CT005232 (Ricke et al. 2014), prior to reassembly of the USCα genomic bin. Annotated ORFs are represented as arrows, colourcoded as indicated by the legend included on the lower left. Subsequences which were aligned between contigs are shown connected by coloured blocks, which are colourcoded based on sequence identity. The USCα Fosmid reference [file EMI-20-1016-s001.pdf]

Supplementary Information:

Bioinformatic analyses of mini-metagenomes and full metagenomes

Read processing: In order to minimize the influence of low quality reads and residual adapter fragments, reads were quality trimmed and adapter clipped using a three-step process, consisting of Trimmomatic (Bolger *et al.*, 2014), bbduk (https://jgi.doe.gov/data-and-tools/bbtools/) and cutadapt (Martin, 2011). Minimum length cutoffs were were set at 45 bp for HiSeq and NextSeq datasets (MFS1, MFS3_1 and MFS3_2), but at 105 bp for MiSeq datasets (MFS2 and single cell datasets). For Trimmomatic the remaining settings were as follows: "ILLUMINACLIP:Trueseq3_PE.fa:2:30:10 LEADING:3 TRAILING:3 SLIDINGWINDOW:4:15". The basic bbduk settings were "-ktrim=2 -minK=11 entropy=0.25". For the final clipping step, the adapter subsequences "AGATCGG" and "CCGATCT" were provided as arguments to cutadapt. Overlapping read pairs were then identified and merged using Flash (Magoč and Salzberg, 2011) using a minimum overlap of 40 bp and a maximum mismatch fraction of 0.15. Single cell datasets were additionally decontaminated by mapping the reads against common MDA contaminants such as E.coli, Homo sapiens, pET-mod and UniVec vectors in addition to PhiX174, using fastq_screen v.0.4.4 (https://www.bioinformatics.babraham.ac.uk/projects/fastq_screen/).

Metagenome assembly: In order to reduce dataset complexity and assembly memory requirements, metagenomic datasets were partitioned into low (<=10x), medium (10-50x) and high coverage (>50x) read fractions based on each reads average k-mer coverage at k=31 using bbnorm (https://jgi.doe.gov/data-and-tools/bbtools/). Preliminary bowtie2 (Langmead and Salzberg, 2012) mappings indicated that the USCα fosmid sequence was exclusively present in the low coverage fraction of both, datasets MFS1 and MFS2. Therefore, in order to minimize k-mer coverage gaps for the targeted USCα assembly, the respective read fractions of MFS1 and MFS2 were combined prior to assembly, resulting in fractions "MFS12_low", "MFS12_mid" and "MFS12_high". Likewise, the respective fractions of datasets MFS3_1 and MFS3_2 were combined to "MFS3_low", "MFS3_mid" and "MFS3_high", since both datasets contained USCα fosmid related reads exclusively in the "medium" coverage fraction. Each fraction (low, medium and high) of each pooled dataset (MFS12 and MFS3) was assembled individually using Metaspades v3.10 (Nurk *et al.*, 2016) using k-mer steps ranging from k=21 to k=121 in steps of 10, as this assembler was shown to produce the best assembly performance for low abundant community members (Vollmers *et al.*, 2017b). Subsequently, the assembled contigs of the different read fractions were combined for each pooled dataset MFS12 and MFS3 by concatenating the respective fasta files after all contigs shorter than 500 bp were removed.

Assembly of FACS based mini-metagenomes: Due to the non-uniform read coverage of MDA products, FACS based mini-metagenomes were assembled individually using Spades v3.10 (Bankevich *et al.*, 2012) in single cell and "careful" mode, with k-mers ranging from 21-121 in steps of 10. In order to remove potential residual MDA contaminants not represented by the previous read screening databases, all metagenomic datasets were mapped onto the mini-metagenome assemblies using bowtie2. Mini-metagenome contigs which were not covered by any metagenomic read were removed as potential contaminants.

Metagenome assembly optimization: In order to maximize contig sequence information for subsequent binning purposes, USCα-enriched mini-metagenome assemblies were combined and subsequently merged with each metagenome

assembly using Minimus2 (Sommer *et al.*, 2007) in directional mode with the mini-metagenomes as subjects and each metagenome assembly as references. Minimum overlap size was set at 500 bp, with a 94% sequence identity cutoff and a maximum of 20 bp divergence allowed at the contig ends. Thereby, the median contig size could be increased more than four-fold for a total subset of 972 potentially USCα enriched original metagenome contigs. This included a total subset of 384 metagenome contigs, which were originally shorter than 1 kb, but reached lengths suitable for subsequent binning steps after merging. Furthermore, a total of 229 additional contigs could be added to the assembly, which were shown to be present in the metagenomes by read mappings, but not successfully reconstructed in the original metagenome assemblies due to low coverage and high complexity. The resulting SCG-merged metagenomes were then similarly merged, using MFS12 as subject and MFS3 as reference.

Contig classification and quantification: 16S and 23S rRNA gene sequences were predicted using rnammer v1.2 (Lagesen *et al.*, 2007) and classified against the SINA database using the least common ancestor approach implemented by SINA (Pruesse *et al.*, 2012). Total proteins were predicted using prodigal v.2.6.3 (Hyatt *et al.*, 2010) and universal single copy marker genes were extracted using fetchMG (Mende *et al.*, 2013). Contigs were then classified, both on total protein and on universal marker level, by BLASTing the respective protein sequences against the NCBI nr database, associating the hits of each protein directly with the respective contig, sorting the results based on alignment score and finally submitting this information to the blast2lca script included with MEGAN6 (Huson and Weber, 2013) distributions. Final taxonomic assignments were selected hierarchically: Whenever possible, rRNA based classifications were preferred over protein-based classifications. Otherwise, marker based classifications were preferred over total protein based classifications. Furthermore, contigs were quantified by mapping each read dataset individually against the final merged assembly using bwa (Li and Durbin, 2010) and calculating the "outlier pileup coverage" using bamm (http://ecogenomics.github.io/BamM/).

Binning: In order to increase binning specificity, the assembly was again partitioned into separate fractions, analoguous to the previous read partitioning, based on relative contig abundances in datsets MFS1 and MFS2 (MFS12) compared to MFS3_1 and MFS3_2 (MFS3). This was considered more specific than the original read partitioning step, as it is based on greater sequence context. Contig partitioning resulted in nine fractions, ranging from MFS12low_MFS3low to MFS12high_MFS3high. Each partition was then binned with MaxBin v2.2.3 (Wu *et al.*, 2016), using the contig coverage information of each read dataset in order to enable a differential coverage based approach.

Analyses and processing of metagenomic bins: Estimations of bin completeness and potential contamination were obtained using the "lineage_wf" workflow of checkM v1.0.4 (Parks *et al.*, 2015). All bins were further "purified" using a two-step approach. First, the predominant taxa were determined for each bin on domain, phylum, class, order and family level, based on the previously calculated, SINA- and blast2lca-based, hierarchical contig classifications. However, for this purpose, classification confidence values produced by blast2lca were also considered: Taxonomic assignments with confidence values below 50 were ignored and assumed "unassigned" instead. All contigs that were confidently classified to a taxon other than "unassigned" and differed from the respective bin's predominant taxon on any level, were removed from the bin. Second, the remaining contigs of each bin were filtered using a z-score based differential coverage approach previously described (Vollmers *et al.*, 2017a) using a custom python script

(https://github.com/jvollme/bin_polisher). Contigs displaying z-score differences of more than 2 between any read datasets were removed.

Identification and reassembly of USCα among metagenome bins: Processed bins were screened for presence of the USCα fosmid sequence using BLAST. Hits were retained if they showed a sequence identity >99.9% over >90% of the alignable subject contig length (but allowing for overhangs extending beyond the fosmid boundaries). Reads corresponding to contigs of a potential USCα bin were extracted from metagenome read mappings using bamm, and reassembled using spades v.3.10 with "careful" mode and automatic k-mer selection. Contigs shorter than 1 kb were removed from the final assembly.

Four contigs corresponding to the USCα fosmid could be identified (Suppl. Fig. S4). The majority (~90%) of the USCα fosmid sequence (Ricke *et al.*, 2005), including a partial *pmoC* gene, could be reconstructed, and even extended, by only two contigs found exclusively in bin "007" of metagenome fraction "*MFS12low_MFS3mid*", which was classified as closely related to the genus *Methylocapsa* within the *Beijerinckiaceae* (Suppl. Fig. S2 and Suppl. Table 1) and subsequently designated as "USCα genomic bin". This metagenome fraction also contained two almost identical contigs, mid_NODE_24546 and mergemg_128398, encoding the remaining USCα *pmoCAB* gene cluster, but these were not unambiguously assigned to any specific bin, most likely due to the dissemination of related *pmo* genes among phylogenetically diverse organisms (Gilbert *et al.*, 2000). However, based on best BLAST hits against the NCBI nr database, both contigs were found to be most closely related to reference sequences of the genus *Methylocapsa,* thereby matching the taxonomic assignment of the USCα genomic bin. The fact that the *pmo* gene cluster sequence was identical between both contigs, with differences only occurring at the extreme contig ends, may indicate strain variations in the exact genomic location of this feature within USCα genomes. Therefore, the relative abundance profiles of the USCα *pmoCAB* containing contigs were tested for compatibility with the USCα genomic bin, by temporarily adding them and performing the z-score based differential coverage filtering approach already used during preliminary bin processing (this study and Vollmers et al, 2017a). Only one of the contigs, mergemg_128398, passed this filtering criterion, thereby proving a matching coverage profile across all metagenomic samples. As a result, and in consideration of the matching taxonomic assignments and co-localization within the known USCα fosmid sequence, this contig was transferred to the USCα genomic bin.

Multilocus sequence analyses: All reference comparison genomes were downloaded from NCBI. In order to maximize the available sequence information for phylogenetic analyses, a custom MLSA workflow was performed: Potential homologs between all reference genomes were detected using the bidirectional BLAST approach implemented by proteinortho5 (Lechner *et al.*, 2011) with the following settings: "-ident=25 -cov=60 -e=1e-8 --selfblast". Single copy core genes were identified based on the proteinortho results and aligned using muscle (Edgar, 2004). In order to remove unalignable N- and C- terminal overhangs, as well as reduce the influence of spurious ORF-calling, each core gene alignment was cropped to the region between the first and last alignment positions that contained sequence information for all aligned gene products. The cropped alignments of all core genes were concatenated and any remaining unalignable regions were filtered using Gblocks (Castresana, 2000) with the following settings: '-t=p -d=n -b3=10 -b4=5 -b5=a' and the '-b2' argument set to 50% of the number of comparison genomes. The complete workflow was implemented in the form of a custom python script (https://github.com/jvollme/PO_2_MLSA). Phylogenetic clustering of the resulting

alignment was performed using fasttree2 (Price *et al.*, 2010) in double precision mode.

Percentage of conserved proteins (POCP) Analyses: In order to better match the criteria described by Qin et al (Qin *et al.*, 2014), proteinortho-based homolog detection was repeated with the following settings: "-ident=40 -cov=50 -e=1e-5 –selfblast". Pairwise POCP values were calculated from the proteinortho results using a custom python script (https://github.com/jvollme/po2pocp).

References:

Bankevich A, Nurk S, Antipov D, Gurevich AA, Dvorkin M, Kulikov AS, *et al.* (2012). SPAdes: a new genome assembly algorithm and its applications to single-cell sequencing. *J Comput Biol* **19**: 455–77.

Bolger AM, Lohse M, Usadel B. (2014). Trimmomatic: a flexible trimmer for Illumina sequence data. *Bioinformatics* **30**: 2114–2120.

Castresana J. (2000). Selection of Conserved Blocks from Multiple Alignments for Their Use in Phylogenetic Analysis. *Mol Biol Evol* **17**: 540–552.

Edgar RC. (2004). MUSCLE: multiple sequence alignment with high accuracy and high throughput. *Nucleic Acids Res* **32**: 1792–7.

Gilbert B, McDonald IR, Finch R, Stafford GP, Nielsen AK, Murrell JC. (2000). Molecular analysis of the pmo (particulate methane monooxygenase) operons from two type II methanotrophs. *Appl Environ Microbiol* **66**: 966–975.

Huson DH, Weber N. (2013). Microbial community analysis using MEGAN. *Methods Enzymol* **531**: 465–85.

Hyatt D, Chen G-L, LoCascio PF, Land ML, Larimer FW, Hauser LJ. (2010). Prodigal: prokaryotic gene recognition and translation initiation site identification. *BMC Bioinformatics* **11**: 119.

Lagesen K, Hallin P, Rodland EA, Staerfeldt H-H, Rognes T, Ussery DW. (2007). RNAmmer: consistent and rapid annotation of ribosomal RNA genes. *Nucleic Acids Res* **35**: 3100–3108.

Langmead B, Salzberg SL. (2012). Fast gapped-read alignment with Bowtie 2. *Nat Methods* **9**: 357–359.

Lechner M, Findeiss S, Steiner L, Marz M, Stadler PF, Prohaska SJ. (2011). Proteinortho: detection of (co-)orthologs in large-scale analysis. *BMC Bioinformatics* **12**: 124.

Li H, Durbin R. (2010). Fast and accurate long-read alignment with Burrows-Wheeler transform. *Bioinformatics* **26**: 589–595.

Magoč T, Salzberg SL. (2011). FLASH: fast length adjustment of short reads to improve genome assemblies. *Bioinformatics* **27**: 2957–63.

Martin M. (2011). Cutadapt removes adapter sequences from high-throughput sequencing reads. *EMBnet.journal* **17**: 10.

Mende DR, Sunagawa S, Zeller G, Bork P. (2013). Acurate and universal delineation of prokaryotic species. *Nat Methods* **10**: 881–4.

Nurk S, Meleshko D, Korobeynikov A, Pevzner P. (2016). metaSPAdes: a new versatile de novo metagenomics assembler. http://arxiv.org/abs/1604.03071

Parks DH, Imelfort M, Skennerton CT, Hugenholtz P, Tyson GW. (2015). CheckM: assessing the quality of microbial genomes recovered from isolates, single cells, and metagenomes. e-pub ahead of print, doi: 10.7287/peerj.preprints.554v2.

Price MN, Dehal PS, Arkin AP, Rojas M, Brodie E. (2010). FastTree 2 – Approximately Maximum-Likelihood Trees for Large Alignments Poon AFY (ed). *PLoS One* **5**: e9490.

Pruesse E, Peplies J, Glöckner FO. (2012). SINA: accurate high-throughput multiple sequence alignment of ribosomal RNA genes. *Bioinformatics* **28**: 1823–9.

Qin Q-L, Xie B-B, Zhang X-Y, Chen X-L, Zhou B-C, Zhou J, *et al.* (2014). A proposed genus boundary for the prokaryotes based on genomic insights. *J Bacteriol* **196**: 2210–5.

Ricke P, Kube M, Nakagawa S, Erkel C, Reinhardt R, Liesack W. (2005). First genome data from uncultured upland soil cluster alpha methanotrophs provide further evidence for a close phylogenetic relationship to *Methylocapsa acidiphila* B2 and for high-affinity methanotrophy involving particulate methane monooxygenase. *Appl Environ Microbiol* **71:** 7472-7482.

Sommer DD, Delcher AL, Salzberg SL, Pop M. (2007). Minimus: a fast, lightweight genome assembler. *BMC Bioinformatics* **8**: 64.

Vollmers J, Frentrup M, Rast P, Jogler C, Kaster AK. (2017a). Untangling genomes of novel Planctomycetal and Verrucomicrobial species from monterey bay kelp forest metagenomes by refined binning. *Front Microbiol* **8**. e-pub ahead of print, doi: 10.3389/fmicb.2017.00472

Vollmers J, Wiegand S, Kaster AK. (2017b). Comparing and evaluating metagenome assembly tools from a microbiologist's perspective - Not only size matters! *PLoS One* **12**. e-pub ahead of print, doi: 10.1371/journal.pone.0169662.

Wu Y-W, Simmons BA, Singer SW. (2016). MaxBin 2.0: an automated binning algorithm to recover genomes from multiple metagenomic datasets. *Bioinformatics* **32**: 605–607.

a

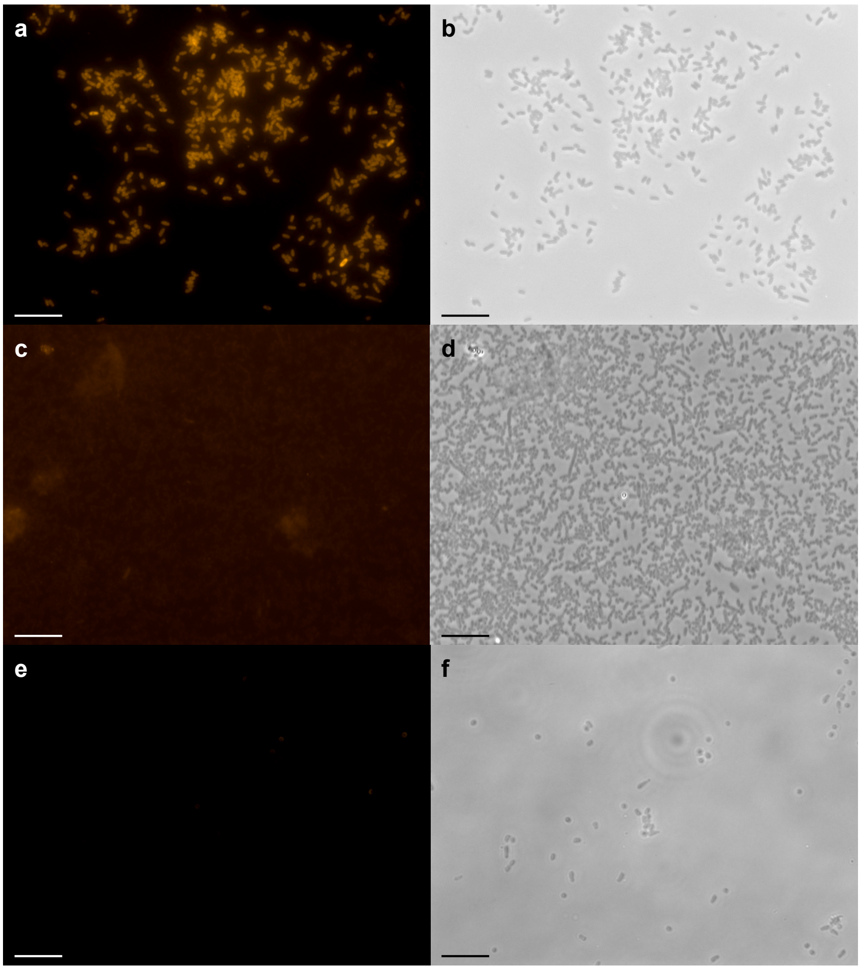

b

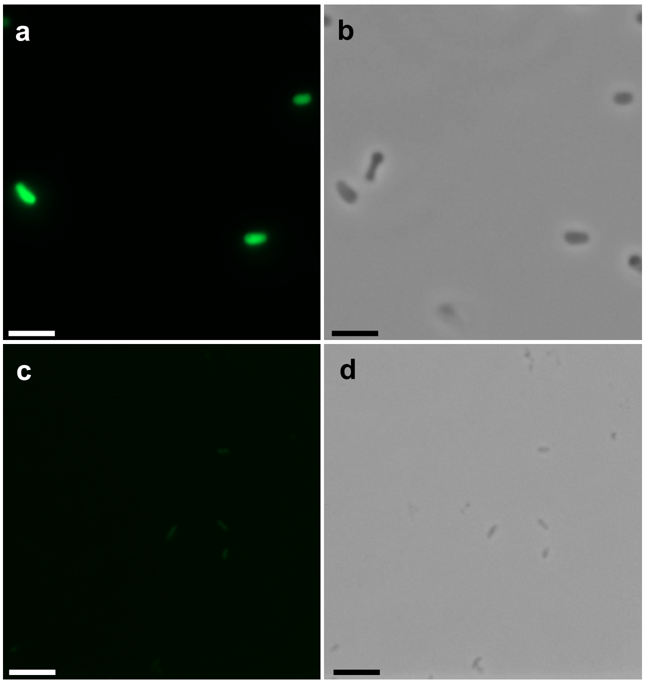

Supplementary Figure S1 | Controls for 16S rRNA DOPE-FISH and pMMO labelling. a, USCα specific 16S rRNA DOPE-FISH (with Cy3 labelled probe MF1) with induced *E.coli* top10 clone expressing the USCα 16S rRNA gene as positive control (a), and non-induced clone (c) and cells of *Methylosinus trichosporium* OB3b (e) as negative controls. b, d, f are respective phase contrasts. Scale bars represent 10 µm. **b,** pMMO labelling by FTCP with active cells of *Methylosinus trichosporium* OB3b as positive control (a) and *Methylovorus* cells as negative control (c). b and d are respective phase contrasts. Scale bars represent 5 µm.

a

b

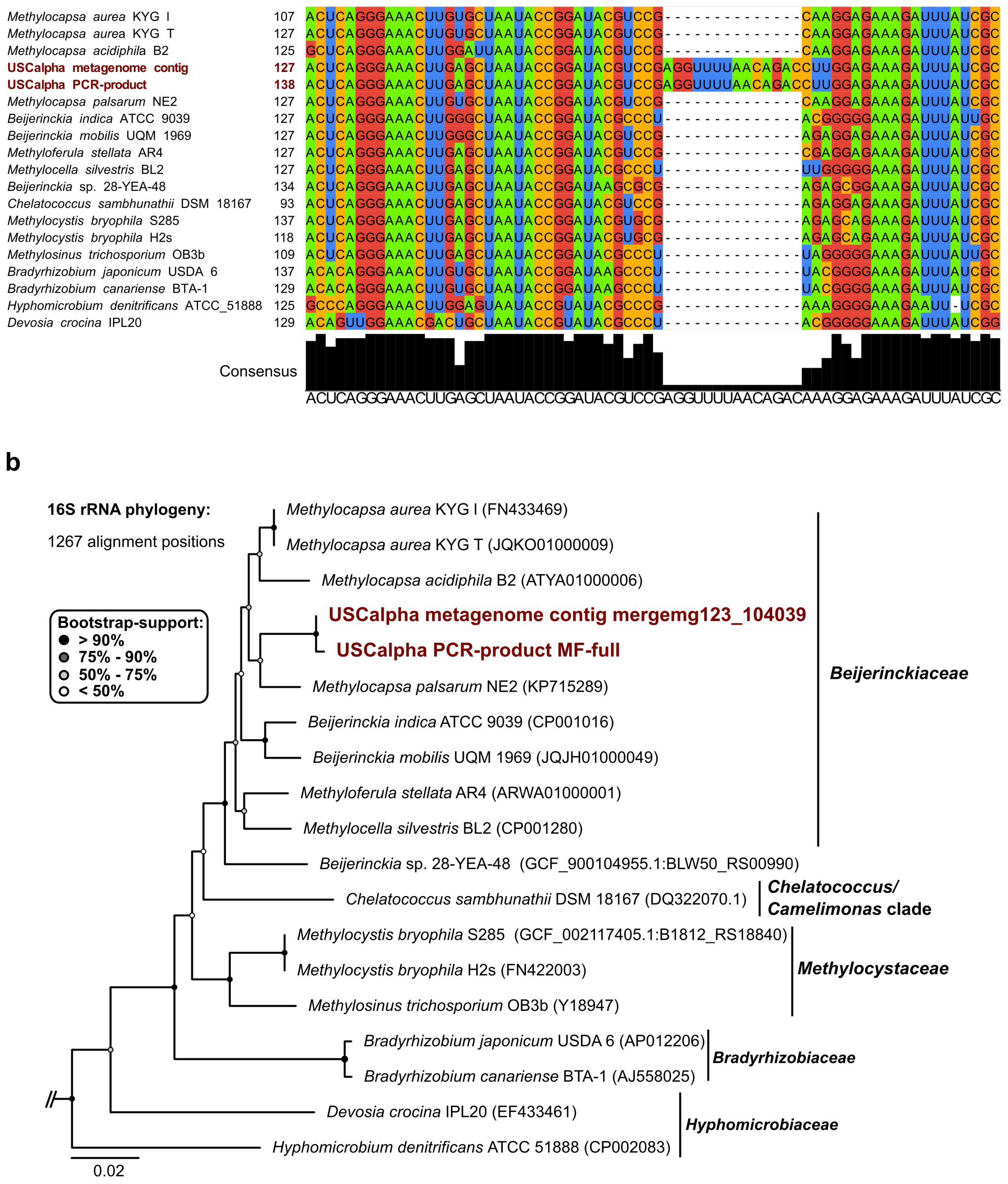

Figure S2 | a, Partial view of 16S rRNA gene alignments showing a short insertion within the USCα 16S rRNA gene and divergence to related *Rhizobiales* sequences. The bottom histogram indicates the conservedness of each alignment position within the comparison strains. **b**, Phylogenetic tree showing the placement of the USCα associated 16S sequences within the *Beijerinckiaceae*. Neighbor-joining tree generated using Arb (Ludwig *et al.*, 2004) based on 1267 shared alignment positions. For each reference sequence, either the respective NCBI assembly accession number or the genome assembly accession number followed by the respective locus tag are given in parantheses. USCα associated sequences are marked in red. Bootstrap support values are indicated by color at each node. *Escherichia coli* KCTC 2441 (accession: EU014689) served as distant outgroup and root (not shown).

	Methylocapsa acidiphila B2	Methylocapsa aurea KYG T	MFS USCalpha	Methylocapsa palsarum NE2	Methylocella silvestris BL2	Beijerinckia indica ATCC 9039	Beijerinckia mobilis UQM 1969	Methyloferula stellata AR4T	Methylocystis bryophila S285	Methylosinus trichosporium OB3b	Beijerinckia sp. 28-YEA-48	Chelatococcus sp. CO-6	Chelatococcus daeguensis M3	Chelatococcus daeguensis TAD1	Chelatococcus sambhunathii DSM 18167	Bradyrhizobium japonicum J5	Bradyrhizobium canariense GAS369	Hyphomicrobium denitrificans ATCC 51888	Devosia crocina IPL20
Methylocapsa acidiphila B2	100%	67%	43%	61%	57%	53%	53%	56%	48%	47%	33%	36%	38%	39%	39%	28%	31%	37%	29%
Methylocapsa aurea KYG T	67%	100%	43%	62%	59%	53%	54%	56%	47%	46%	34%	37%	40%	41%	39%	28%	31%	39%	30%
MFS USCalpha	43%	43%	100%	43%	40%	37%	37%	38%	34%	33%	25%	27%	30%	30%	29%	22%	25%	29%	23%
Methylocapsa palsarum NE2	61%	62%	43%	100%	58%	48%	49%	51%	47%	44%	32%	36%	37%	38%	37%	28%	31%	37%	29%
Methylocella silvestris BL2	57%	59%	40%	58%	100%	51%	53%	56%	43%	43%	34%	36%	38%	39%	38%	30%	33%	39%	29%
Beijerinckia indica ATCC 9039	53%	53%	37%	48%	51%	100%	69%	52%	41%	41%	33%	36%	38%	39%	38%	28%	31%	35%	30%
Beijerinckia mobilis UQM 1969	53%	54%	37%	49%	53%	69%	100%	52%	41%	42%	33%	36%	38%	40%	38%	27%	30%	36%	29%
Methyloferula stellata AR4T	56%	56%	38%	51%	56%	52%	52%	100%	44%	44%	35%	36%	39%	40%	38%	30%	35%	38%	30%
Methylocystis bryophila S285	48%	47%	34%	47%	43%	41%	41%	44%	100%	52%	30%	33%	34%	35%	34%	26%	28%	34%	26%
Methylosinus trichosporium OB3b	47%	46%	33%	44%	43%	41%	42%	44%	52%	100%	31%	34%	35%	36%	36%	26%	28%	35%	27%
Beijerinckia sp. 28-YEA-48	33%	34%	25%	32%	34%	33%	33%	35%	30%	31%	100%	37%	38%	38%	37%	32%	36%	29%	27%
Chelatococcus sp. CO-6	36%	37%	27%	36%	36%	36%	36%	36%	33%	34%	37%	100%	80%	81%	80%	36%	36%	34%	37%
Chelatococcus daeguensis M3	38%	40%	30%	37%	38%	38%	38%	39%	34%	35%	38%	80%	100%	93%	94%	33%	34%	37%	38%
Chelatococcus daeguensis TAD1	39%	41%	30%	38%	39%	39%	40%	40%	35%	36%	38%	81%	93%	100%	92%	33%	35%	38%	39%
Chelatococcus sambhunathii DSM 18167	39%	39%	29%	37%	38%	38%	38%	38%	34%	36%	37%	80%	94%	92%	100%	33%	34%	36%	38%
Bradyrhizobium japonicum J5	28%	28%	22%	28%	30%	28%	27%	30%	26%	26%	32%	36%	33%	33%	33%	100%	56%	25%	23%
Bradyrhizobium canariense GAS369	31%	31%	25%	31%	33%	31%	30%	35%	28%	28%	36%	36%	34%	35%	34%	56%	100%	29%	25%
Hyphomicrobium denitrificans ATCC 51888	37%	39%	29%	37%	39%	35%	36%	38%	34%	35%	29%	34%	37%	38%	36%	25%	29%	100%	31%
Devosia crocina IPL20	29%	30%	23%	29%	29%	30%	29%	30%	26%	27%	27%	37%	38%	39%	38%	23%	25%	31%	100%

Qin Q-L, Xie B-B, Zhang X-Y, et al.(2014) A Proposed Genus Boundary for the Prokaryotes Based on Genomic Insights. Journal of Bacteriology; 196(12):2210-2215. doi:10.1128/JB.01688-14

Figure S3 | Pairwise pocp values. Values above the proposed genus cutoff (Qin *et al.*, 2014) are indicated in red.

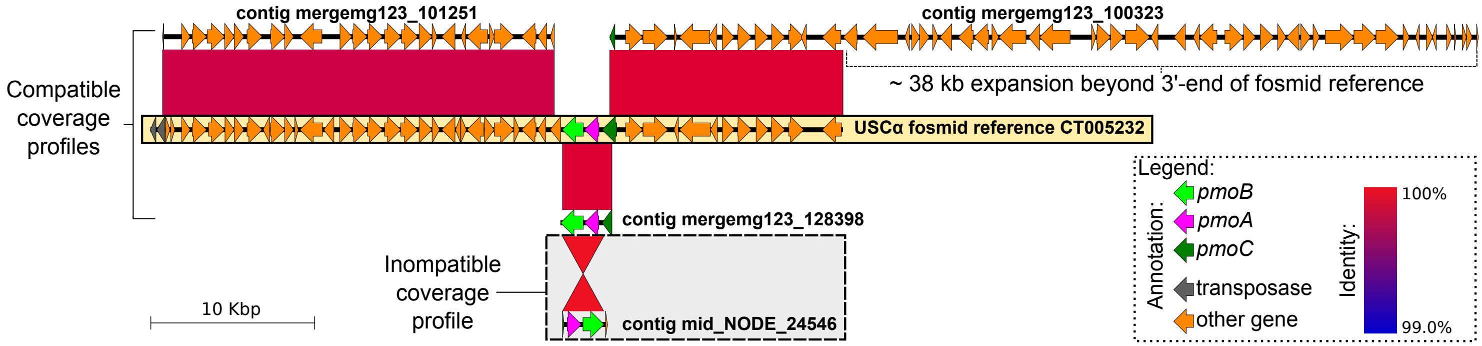

Supplementary Figure S4: Alignment of metagenomic contigs against the USCα fosmid reference CT005232 (Ricke et al. 2014), prior to reassembly of the USCα genomic bin. Annotated ORFs are represented as arrows, colorcoded as indicated by the legend included on the lower left. Subsequences which were aligned between contigs are shown connected by colored blocks, which are colorcoded based on sequence identity. The USCα Fosmid reference could be almost completely reconstructed by only three contigs (mergemg123_101251, mergemg123_100323 & mergemg123_128398) showing >99.9% sequence identity over the complete respective alignment length as well as compatible coverage profiles. In addition, the fosmid reference sequence context was expanded by ~ 38 kb by metagenome contig mergemg123_100323. An similar expansion of the 5'-end of the fosmid reference was not possible, due to the presence of a transposase gene, The USCα signature pmoBAC genecluster was found to be encoded contig mergemg123_128398 as well as contig mid_NODE_24546, sharing 100% sequence identity over the gene cluster but differing at the extreme contig ends, thereby indicating potential strain heterologies concerning the exact genomic location of this gene cluster. However, in contrast to contig mergemg123_128398, the coverage profile of contig mid_NODE_24546 did not prove compatible with the fosmid reference and the respective genomic bin, therefore this contig was excluded from the metagenomic bin.
